# Dogs’ Sociability, Owners’ Neuroticism and Attachment Style to Pets as Predictors of Dog Aggression

**DOI:** 10.3390/ani10020315

**Published:** 2020-02-18

**Authors:** Elena Gobbo, Manja Zupan

**Affiliations:** Department of Animal Science, Biotechnical Faculty, University of Ljubljana, Groblje 3, 1230 Domžale, Slovenia; manja.zupan@bf.uni-lj.si

**Keywords:** dogs, dog owners, aggression, personality traits, attachment

## Abstract

**Simple Summary:**

It is widely known that psychological characteristics, for example personality traits, can facilitate the occurrence of aggressive behavior. Using the combination of two research methods—questionnaires and behavioral testing—we investigated the associations between a dog’s personality and its aggression towards humans and animals. Due to the close relationship and co-habitation of dogs and humans, we also looked at how the owner’s personality and the dog–human emotional bond, known as attachment style, play a role in dog aggression. Our results indicated that dogs which were aggressive towards humans were less sociable, and had owners who were less emotionally stable, more distant, and less clingy and controlling, compared to non-aggressive dogs. These results emphasize the importance of owner attachment to a dog for dog behavior, and may serve as a foundation for future research on psychosocial factors influencing dog aggression.

**Abstract:**

A dog’s aggressive behavior is influenced by external and internal factors, including its psychological profile. In this study, dogs’ and owners’ personalities and the owners’ attachment style to their dogs were identified and associated with owner-reported dog aggression towards humans and animals. Forty Slovenian owners participated with their dogs, of different breeds and aggression history, sorted into three groups (non-aggressive dogs, dogs aggressive towards humans, and dogs aggressive towards animals). The owners filled out three separate questionnaires that assessed dog aggression history towards different targets, owner’s personality and degree of insecure attachment styles to dogs; namely anxious and avoidant attachment. Dog personality was characterized using a standardized dog mentality assessment test, during which the dog was exposed to nine tasks, performed outside, and dogs were scored based on behaviors they exhibited. The results indicated that dogs which were aggressive towards humans were less sociable than non-aggressive dogs and this was associated with the higher neuroticism scores of their owners. We also found that dogs which were aggressive towards strangers had owners with lower scores for anxious attachment and that dogs which were aggressive towards owners had owners with higher scores for avoidant attachment. These results imply that the psychological profiles of both a dog and its owner influence dog aggression towards humans.

## 1. Introduction

Historically, the primary role of dogs was in guarding, herding and hunting, but their high socio-cognitive abilities and capability to form a close relationship with humans [1,2] have made them an integral part of human society. Nowadays, whilst many still play an important role as working dogs [3,4,5,6], the most common reason for owning a dog is companionship [7]. Despite the fact that the role of pet dogs in Western cultures has been elevated to the status of a family member [8], there are several factors that can negatively affect the quality of the dog–human bond, with aggression being the most serious [9,10]. Aggressive behavior of dogs is expressed as aggressive biting, by snapping or attacking, and aggressive threatening, by growling, barking and baring their teeth [11]. It can be classified by motivational basis (territorial-, fear-, possessiveness-related, etc.) or targeted basis (stranger-, owner-, dog-directed etc.) [12], and can be influenced by a variety of factors. These include environment, maternal and sibling interactions, experience in the form of socialization and learning, as well as different biological [13] and psychological correlates, including an individual’s personality traits. 

Animal personality is defined as a consistency of inter-individual behavioral traits through time and across contexts [14,15] and can be characterized using standardized tests [16]. In dogs, there are two main methods for the assessment of personality: questionnaires and behavioral tests [17]. Both methods have advantages and disadvantages. For instance, gaining information from dog owners using questionnaires is less time consuming, allows for a larger and a more diverse sample, and involves a person who lives with the dog and knows more about the dog’s everyday behavior [18]. On the other hand, it means that behavior is often assessed by people that do not have sufficient knowledge of animal behavior. This makes behavioral observations and interpretations made by professionals in the field more objective, precise and free of owner bias [19]. One of the most widely used behavioral tests is the standardized Dog Mentality Assessment (DMA) that was developed by the Swedish Working Dog Association and measures a dog’s reaction to different stimuli [20]. The assessments using the DMA revealed five personality traits, labelled as playfulness, curiosity/fearlessness, chase-proneness, sociability and aggressiveness, as well as one broader dimension named shyness/boldness, that is generalized for the dog as a species [20]. Using questionnaire data, personality traits were found to be associated with potential aggressive behavior. For example, more fearful dogs were associated with dog-directed aggression and fear-related aggression [21,22], while lower levels of sociability were related to higher levels of stranger-directed and child-directed aggression [23].

Aside from the dog’s personality traits, its owner’s psychological characteristics may lead to pronounced dog aggression, due to their co-habitation and close relationship. For instance, owners with lower ratings for the personality traits of agreeableness, emotional stability, extraversion and conscientiousness often have dogs showing higher levels of aggression towards owners and a fear of strangers [24]. Additionally, the owner’s personality traits have also been significantly correlated with those of their dog, using the Big Five factor taxonomy [25]. The reason behind this may be the shared social environment and activities, resulting in a higher degree of emotional contagion, or a selection process, where the owners select a dog that matches their personality and lifestyle, as seen in romantic partner and friend selection in humans [26].

Another factor that may influence a dog’s aggressive behavior is the cognitive-emotional bond, known as attachment. The concept of attachment was initially developed to describe the affectional bond of children to their caregivers and later between adults [27]. The use of the term was further extended into contexts involving humans and objects, places and non-human animals [28,29]. It was previously suggested that humans can form an attachment to their dogs [30] and that this relationship is comparable to the one between a parent and a child [31], as human behavior towards dogs and children tends to be similar [32,33]. The attachment between two individuals can be secure, defined by comfort with intimacy and trust, or insecure. Two examples of insecure attachment are anxious attachment, characterized by clingy, controlling behavior, and avoidant attachment, defined by avoidance of intimacy [34]. A dog–human bond is more influenced by human factors than canine factors [35]; therefore the owner’s attachment style may have an impact on the dog’s behavior. For example, it has been reported that dogs of owners with lower or higher adult attachment scores (in attachment anxiety, confidence and avoidant attachment subscales) may develop different behavioral strategies during challenging situations [36]. Additionally, the adult attachment styles have also been associated with behavioral problems in dogs; more precisely, owners scoring high on avoidant attachment were reported to have dogs with increased occurrence of separation-related disorder [37].

Here, we investigated the associations between owner- and dog-related psychosocial factors and dog aggression towards different targets, using a combination of two research methods: behavioral testing, that has not previously been used while studying dog aggression from a psychosocial perspective, and questionnaire-based evaluations. For behavioral testing, we used the personality taxonomy of Svartberg and Forkman [20]. Based on the previous studies using only questionnaires, we predicted that dogs with owner-reported aggressive behavior would have higher trait scores for aggressiveness and chase-proneness, and lower trait scores for playfulness, curiosity/fearlessness and sociability. Secondly, we hypothesized that aggressive dogs would be associated with owners having lower scores for agreeableness, extraversion and conscientiousness, but higher neuroticism, and higher owner–dog anxious and avoidant attachment scores than non-aggressive dogs.

## 2. Materials and Methods 

### 2.1. Participants

Forty dog–owner dyads participated in the study. To include a sample of dogs with diverse behavioral backgrounds, and to compare dogs with and without behavior problems, owners were asked to report their dogs’ behavioral history before participation in the study. Once the data were collected, dogs were placed into one of the following categories: dogs with no history of aggression (n =14), dogs with a history of aggression towards humans (n = 13), and dogs with a history of aggression towards dogs and other animals (n = 13). Dogs were of both sexes (16 females; 24 males) and were all older than one year (mean age ± SD, 4.1 ± 2.8 years). There were 17 mongrels and the rest were one of 12 breeds: Pekingese, Tibetan Terrier, Karst Shepherd, Border Collie, Australian Shepherd, German Spitz, Entlebucher, Coton de Tulear, Central Asian Shepherd, Shiba Inu, Brittany, Stafford Terrier. None of the dogs had been previously trained for any particular test battery. The owners accompanying the dogs during behavioral testing were primarily female (n = 26, 65%), aged between 19 and 64 years (mean age ± SD, 33.8 ± 12.7 years) and were the dogs’ primary attachment figure (mean years ± SD of cohabitation, 3.9 ± 2.9).

### 2.2. Protocol

The dog owners were contacted through social media, faculty mailing lists and canine clubs. Those willing to participate received an online generated survey (OneClick survey software^©^ 200–2018 University of Ljubljana, Faculty of Social Sciences, Centre for Social Informatics; www.1ka.si) containing demographic questions, a Canine Behavioral Assessment and a Research Questionnaire (C-BARQ) [18], a Big Five Inventory questionnaire (BFI-10) [38] and an Experiences in Close Relationship—Revised questionnaire (ECR-R) [34]. The owners and their dogs afterwards participated in an adapted DMA test [20] that was performed in a secured open field.

### 2.3. Assessment of Dogs’ Aggressive Behaviour

Further information on the dogs’ behavioral history was obtained using the C-BARQ questionnaire for owners [18]. The full questionnaire consists of 68 items, divided into 11 categories, but in this study only aggression related factors (9 items for stranger-directed aggression, 8 items for owner-directed aggression, 3 items for dog-directed aggression and 4 items for chasing) were used. The owners were asked to grade their dogs’ typical behavior in a described situation on a 5-point rating scale. The category on chasing behavior was scored on a 5-point frequency scale (0 = never, 1 = seldom, 2 = sometimes, 3 = usually, and 4 = always). For categories regarding owner-, stranger- and dog-directed aggression, a 5-point qualitative scale was used (0 = no signs of the behavior, 1 to 3 = mild to moderate signs of the behavior, and 4 = severe signs of the behavior). As suggested by Hsu and Serpell [18], a brief description of mild, moderate and severe signs of aggression was included in the questionnaire before every question, to help the responder with the grading of their dog’s behavior. The mean value of all answers within each category presented the final score of the category, with the higher score representing a more severe expression of behavior.

### 2.4. Personality Assessment of Dogs

**Figure 1:**
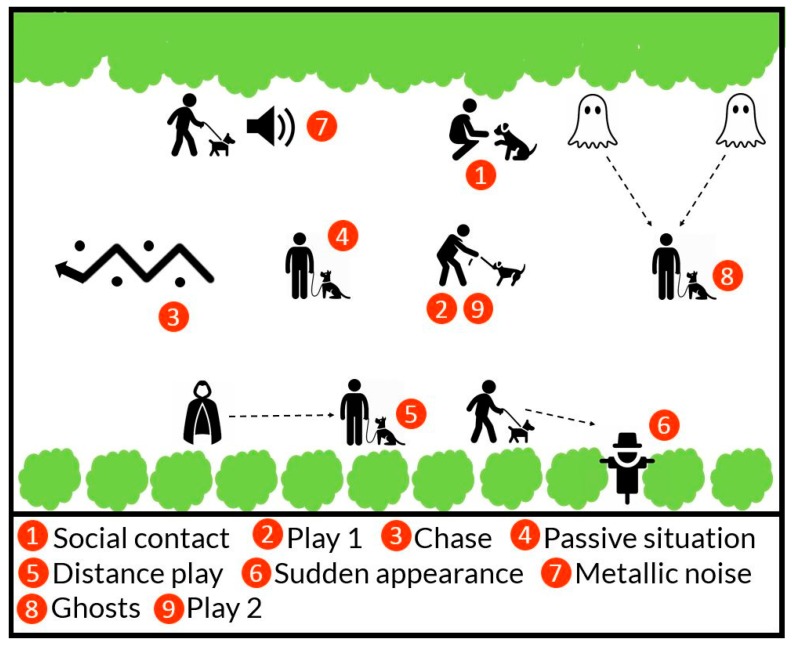
Overview of the outdoor testing area and the position of each subtest; (1) Social contact, (2) Play 1, (3) Chase, (4) Passive situation, (5) Distance play, (6) Sudden appearance, (7) Metallic noise, (8) Ghosts, (9) Play 2.

The DMA behavior test was used to determine the dogs’ personality traits. The behavioral test consisted of nine subtests that were performed outside in a specifically set test area built in advance (Figure 1). Originally there were 10 subtests, but we excluded the last subtest called Gunshot, because shooting a gun was prohibited at the test location. In addition to the owner that accompanied the dog during the testing, three other persons were present—a test leader, an observer and an assistant. The test leader instructed the owner on how to act before and during each subtest and led the owner through the test. The observer video recorded the dogs’ behavioral responses in the test using a Canon XA20 Camcorder. The assistant performed tasks such as pulling up the dummy during the Sudden Appearance subtest. The equipment and its installation was the same in all tests to ensure that the test conditions were similar for the dogs. For safety reasons, dogs were secured with a long (9 m) training leash, even while released from a tighter grip. All the dogs completed the test without any breaks, with a duration of approximately 30 min for each dog. 

After the completion of the testing, the dogs’ behavioral responses were coded and scored. Score sheets contained subtests, predefined behavioral variables and descriptions of behavior for score 1, 3 and 5 (Table S1). The behavior descriptions for scores 1 and 5 were as described by Svartberg and Forkman [20], while for score 3 we added our own descriptions. A low or high score represented a low or high intensity of the dog’s reaction. Based on the scores of behavioral variables, trait scores for each individual dog were calculated (see Svartberg et al., [38]). A second independent person conducted an inter-rater reliability scoring on 30% of the videos. Consistency between coders using an intra-class correlation coefficient (ICC) was excellent: ICC (consistency) >0.9.

### 2.5. Psychological Assessment of Owners

The abbreviated version of the Big Five Inventory, BFI-10 [39] was used to assess the personality traits of the owners. BFI-10 measured the components of the five factors defined as extraversion, agreeableness, conscientiousness, neuroticism and openness. It consisted of 10 items describing statements about personality, rated on a 5-point scale (1 = disagree strongly, 2 = disagree a little, 3 = neither agree nor disagree, 4 = agree a little, and 5 = agree strongly), with two items for each factor. One item in each factor was reverse scored. The mean value of both answers within each factor represented the final score of the factor 2.6. Owners’ Attachment Styles.

A modified ECR-R [34], based on the ECR-R for humans [40], was used to assess owner attachment styles to dogs. Eight items regarding pet-related anxiety and eight items regarding pet-related avoidance were rated on a 7-point scale (1 = strongly disagree to 7 = strongly agree). The mean value of all answers within each variable represented the final score of the attachment style, with a higher score presenting the more severe expression of pet-related anxiety and avoidance.

### 2.6. Ethical Note

The study was conducted in accordance with the Administration of the Republic of Slovenia for Food Safety, Veterinary Sector and Plant Protection (U3440-14/2019/15). The owners signed a form consenting to data usage and videotaping of the experiment and were given the right to withdraw from the study at any time if the dog showed signs of distress or without giving any reason.

### 2.7. Statistical Analysis

Data were analyzed using SAS 9.4 (SAS Institute, Cary, NC, USA). Dogs were assigned to three groups, based on their owners’ report, (1) non-aggressive group, (2) aggressive towards humans group and (3) aggressive towards animals group. A general linear model (GLM) analysis was used to assess the differences between groups. The residuals followed a normal distribution. For the dogs’ personality traits (playfulness, curiosity/fearlessness, chase-proneness, sociability, aggressiveness, shyness/boldness), the fixed effect of the group was tested for differences and the effect of the dogs’ age was tested as a covariate. For the owners’ personality traits (extraversion, agreeableness, conscientiousness, neuroticism and openness) and the owners’ attachment styles (anxious and avoidant), the fixed effects of the group and the owners’ gender were tested for differences and the effect of the owners’ ages was tested as a covariate. Statistical significance was accepted if *p* < 0.05 and tendency if *p* < 0.10. When a significant effect was found, the LSMEANS and ESTIMATE statements were used to estimate the contrasts between factor levels and to compare their means. When more than two means needed to be compared, a multiple post-hoc Tukey–Kramer test was utilized to find the significant differences. Pearson correlation coefficient calculations were performed using the proc CORR to assess the relationship between the attachment styles and the dogs’ personality traits, the owners’ personality traits, and the dogs’ aggressive behavior, also separately for each of the aggressive classification groups. In the text, only Bonferroni-corrected statistically significant values (*p* ≤ 0.05, B: *p* ≤ 0.01) and coefficients >0.6 are reported. Four participants did not fill out the ECR-R questionnaire regarding attachment styles and their responses were considered as missing data.

## 3. Results

**Figure 2:**
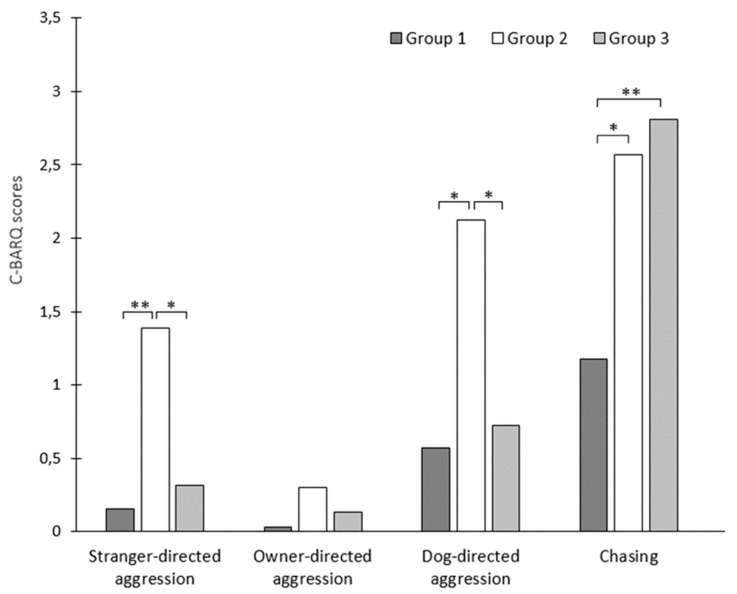
Owner-reported dog behavior using the C-BARQ questionnaire scoring by aggression groups (* *p* < 0.05; ** *p* < 0.001). Group 1 = non-aggressive dogs; Group 2 = dogs, aggressive towards humans; Group 3 = dogs, aggressive towards animals.

The dogs were placed in one of three groups (non-aggressive dogs, dogs aggressive towards humans and dogs aggressive towards animals) based on aggression history reported by their owner. Using behavioral data from C-BARQ, we found that dogs of different groups differed in stranger-directed aggression (F = 10.0, *p* < 0.001), dog-directed aggression (F = 8.71, *p* < 0.001) and chasing (F = 6.02, *p* < 0.001) (Figure 2). Dogs classed as aggressive towards humans had the highest scores for stranger-directed aggression and dog-directed aggression, while both classes of aggressive dogs had higher scores for chasing compared to non-aggressive dogs. Non-aggressive dogs had the lowest scores in all four categories.

Looking at the personality assessment of dogs derived from the DMA, sociability was the only trait which differed statistically between the groups (F = 4.5, *p* = 0.02) (Table 1). Non-aggressive dogs had higher sociability scores compared to dogs aggressive towards humans (*p* < 0.01). The age of the dogs was found to have an effect on their personality traits. Older dogs were less playful (F = 17.54, *p* = 0.0002), less chase-prone (F = 8.91, *p* < 0.005) and more shy in the shyness/boldness dimension (F = 12.14, *p* < 0.001) than younger dogs.

The owners’ personality assessment revealed neuroticism as the only statistically different trait between the dog aggression groups (Table 2). Owners of dogs which were aggressive towards humans had higher scores for neuroticism compared to other owners (both comparisons *p* < 0.05). The gender (F = 5.62, *p* < 0.02) and age of the owner (F = 4.81, *p* < 0.04) was found to have an effect on the owners’ personality traits. Male and older owners were less extraverted than females and younger owners. The younger owners had higher scores for openness than the older owners (F = 9.78, *p* < 0.004).

Owners’ attachment styles did not differ between the groups (avoidant attachment: F = 0.38, *p* = 0.54; anxious attachment: F = 1.88, *p* = 0.17). However, a correlation analysis revealed that dogs of owners with higher scores for anxious attachment were less aggressive towards strangers, more sociable and had lower scores for chasing (Table 3). Those owners whose scores for avoidant attachment were higher had lower scores for conscientiousness and owned dogs with higher scores for owner-directed aggression.

**Figure 3:**
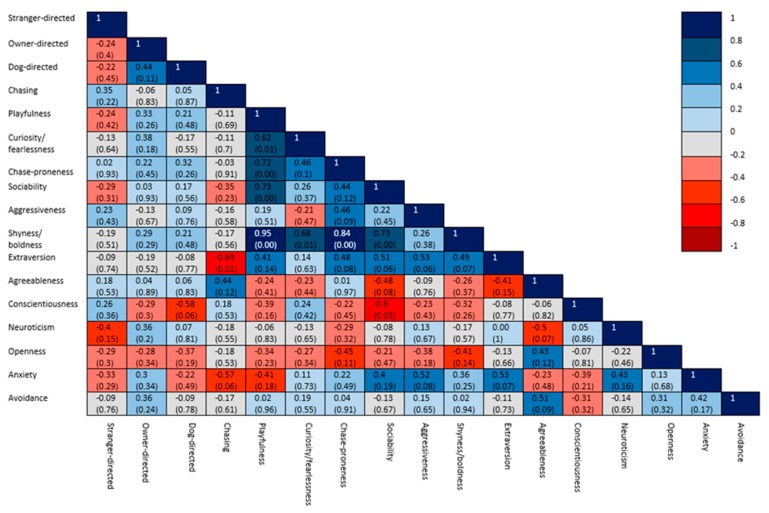
Correlation matrix of the coefficients of the variables in the group of non-aggressive dogs, with significance levels in brackets.

**Figure 4:**
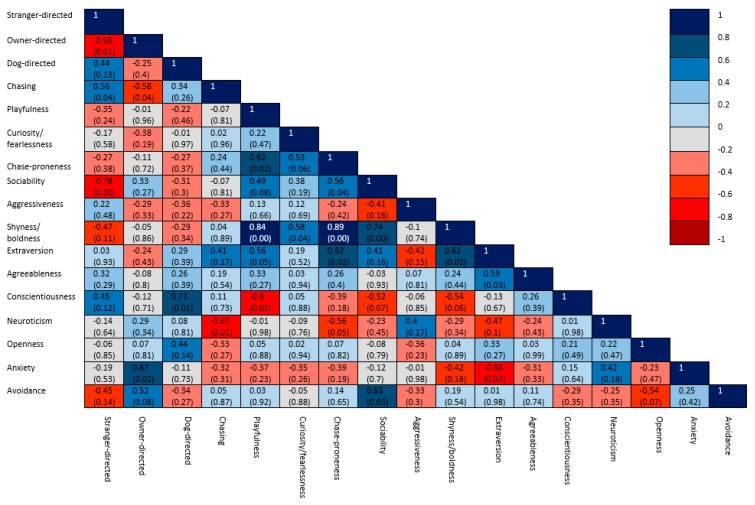
Correlation matrix of the coefficients of the variables in the group of dogs which were aggressive towards humans, with significance levels in brackets.

**Figure 5:**
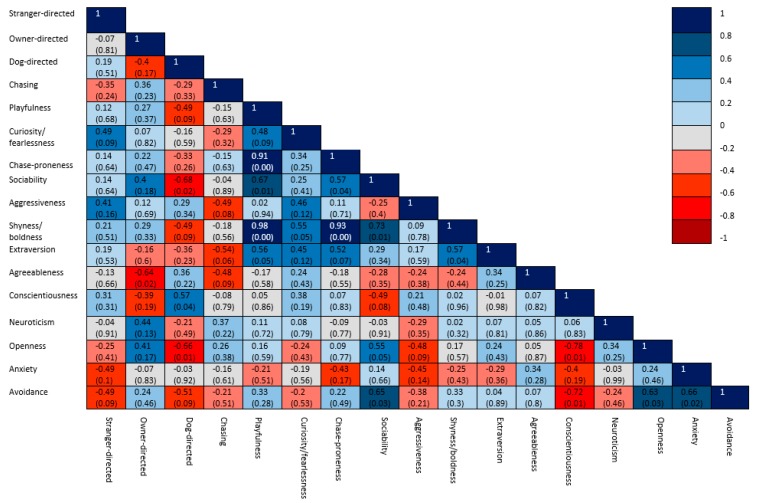
Correlation matrix of the coefficients of the variables in the group of dogs which were aggressive towards dogs and other animals, with significance levels in brackets.

Within each of the aggression groups, significant correlations were found between the observed variables (Figure 3, Figure 4 and Figure 5). In the group of non-aggressive dogs (Figure 3), more extraverted owners had dogs with lower scores for chasing behavior. More playful dogs were more sociable, chase-prone and fearless. In the group of dogs which were aggressive towards humans (Figure 4), dogs with higher stranger-directed aggression were less sociable and less aggressive towards the owner. More neurotic owners were associated with dogs expressing a higher level of chasing behavior. In the group of dogs which were aggressive towards dogs and other animals (Figure 5), dogs with more expressed dog-directed aggression had less open owners. More conscientious owners were found to be less open and had lower scores for avoidant attachment and more playful dogs were found to be more chase-prone.

## 4. Discussion

Using a combination of behavioral testing of the dog, and owner-reported questionnaires, our findings show that dog and owner personality profiles were strongly associated with dog aggression. Dogs classed as aggressive towards humans were found to be less sociable and had owners with higher scores for neuroticism. Our main results also reveal a previously unreported relationship between an owner’s insecure attachment style to a dog and dog aggression. We showed that high avoidant attachment of owners was associated with high levels of owner-directed aggression, while a high anxious attachment was associated with low levels of stranger-directed aggression. 

When examining the relationships between dog personality scores and owner-reported dog aggressive behavior, sociability was the only personality trait associated with the behavior. Dogs classified as aggressive towards humans had lower sociability scores than non-aggressive dogs. Furthermore, within this group, a correlation analysis revealed that less sociable dogs were more aggressive towards strangers. This is in line with previous findings showing that high scores for sociability are linked to lower levels of stranger-directed and child-directed aggression in dogs [23]. It seems that sociable dogs are more comfortable around strangers and in new environments, resulting in lower stress levels and a better social control that may reduce aggressive responses [41]. A personality trait positively associated with sociability in our study was playfulness, but only in the group of non-aggressive dogs. Finding this association only in the group of non-aggressive dogs implies that the social evolutionary purpose of play is a normal social behavior. The function of social play is to enable a more flexible development of future behaviors and a better socio-cognitive development [42,43] with the improvement of communication skills and social ties [44,45]. Play may thus contribute an important role in the appropriate (non-aggressive) social behavior of dogs. Furthermore, playfulness was also positively associated with chase-proneness in the groups of non-aggressive dogs and dogs which were aggressive towards animals, which was previously reported by Svartberg [46] while validating dog behavioral traits. 

When considering the owners’ personality traits, neuroticism was the trait found to have the main impact on the manifestation of dogs’ behavioral problems. The dogs of more neurotic owners were characterized as being the most aggressive of all the dogs towards both strangers and dogs. In the group of dogs classified as showing aggressive behavior towards humans, high owner scores for neuroticism were associated with more prominent chasing behavior in the dog. The reason behind this may be found in the fact that owners’ neuroticism may affect the social behavior of their pet, causing behavioral problems and/or aggressive behavior [47]. A second relevant, although less influential, personality trait was conscientiousness. In the group of dogs classified as showing aggressive behavior towards animals, owners scoring high on conscientiousness were less open. The less open owners were younger individuals who had dogs with higher levels of owner-directed aggression. More conscientious individuals tend to be more organized, responsible and self-disciplined [48], which possibly makes them more controlling, leading to a dog which is less prone to engage in play with a stranger. The other explanation could be that highly conscientious and neurotic individuals tend to prefer dog breeds they perceive as more aggressive [49]. Looking at the owners’ neurotic personality trait, similar positive associations to those found in our study between neurotic owners and behavioral problems, such as the aggression of pets, have been reported in another dog study [50], and in cats [47], as well as in humans [51]. According to Schöberl et al. [52], who suggested that neurotic individuals have a higher stress level based on higher cortisol levels, and Finka et al. [47] recently claimed that neurotic owners affect their pets’ behavior by being less warm, more hostile and overall displaying unpredictable styles of caretaking, resulting in higher stress levels and decreased social control of their cat. We may therefore argue that these neurotic dog owners have a specific behavioral and physiological profile that affects their pets. Another relevant personality trait, however seen only in the non-aggressive dog group, was extraversion, with more extraverted owners, mostly younger females, having dogs with lower levels of chasing. As extraverted people are more inclined to attend various social events and activities, socialize and enjoy the company of other people [53], it is possible that they include their dogs in these activities, making dogs more socialized to other people and animals, and more comfortable in new environments, which might have resulted in a lower level of chasing. 

The finding that owners with higher scores for neuroticism were associated with aggressive dogs partly supports our hypotheses. We based our hypotheses on the study by Dodman et al., [24], where 1564 people responded to an online battery of questionnaires and where it was concluded that owners with lower scores of extraversion, agreeableness, conscientiousness and emotional stability (high neuroticism) own dogs which are more susceptible to develop owner-directed aggression. Although our sample size may have limited the ability to detect potential associations between owners’ personality traits and dog behavior, adding behavioral testing of dogs to objectively assess aggression-related traits enabled our data to be without possible owner biases, and thus more reliable. Questionnaires are known to be a less reliable, less objective method of assessment, although they are less time consuming to administer [19]. It may thus not be surprising to see differences between our results and those in literature, because all previous studies were done using questionnaires only [25,54,55], or even the same Big Five factor taxonomy in both dog and owner assessments [25], just to be able to easily compare dyad scores.

In this study we are first to confirm a relationship between owners’ attachment style to pets and dog aggression. Before discussing this further, it is important to note that we assessed the owners’ attachment to their dogs, while in other studies mentioned below, the adult attachment styles to other people were used for studying the relationship between owners’ attachment and their pet’s behavior. We showed that both degrees of the insecure attachment styles, anxious and avoidant, play a role in dog aggression, regardless of a dog being identified by their owner as a non-aggressive dog, a dog aggressive towards humans, or a dog aggressive towards animals. The owners whose scores for avoidant attachment were higher had lower scores for conscientiousness and owned dogs with higher scores for owner-directed aggression. This can partly be explained by the idea that a more avoidant attachment style to pets might influence the owner’s behavior toward their dog as they distance themselves from the dog, being ignorant and not providing enough affection, intimate contact and availability, as seen in adult attachment [56]. As a result, the dog may perceive a lack of consistent responsiveness to its needs as an indication that it cannot use its owner as a secure base, as it was previously suggested that owners can represent a secure base for their dogs [57], especially in a threatening situation [36,58]. This might evoke fear in dogs, which is one of the most common motivations for aggressive behavior [10]. Security gained from a caregiver may reduce or eliminate the level of fear in dogs. Similar behavioral responses to those we found have been reported in children of parents with a more avoidant attachment style. Children tended to be less attentive toward their parents [59] and more distressed [60] during a stressful event.

On the other hand, and contrary to our hypotheses, dogs with higher scores for stranger-directed aggression were associated with owners who had lower scores for anxious attachment to pets. It seems that highly anxious attachment behavior of the owner, such as constant seeking of support and closeness, clinginess and controlling behavior [61], does not promote aggression. This is in contrast to studies in humans, where it has been reported that anxious mother–infant attachment increases the risk of child aggression [62]. It also seems that anxiety does not contribute to the lack of responsiveness [63] seen among people scoring higher in avoidant attachment, which can lead to a more stressful situation for the dog and potentially facilitate aggression. We also found correlations between owner attachment style to pets, and dogs’ and owner’s personality traits, as seen in human adults [64]. Contrary to our findings showing that more conscientious dog owners were associated with higher scores for avoidant attachment to dogs, Carver [64] found an association with extraversion, agreeableness and neuroticism. A further strong correlation was found between dog owners with high scores for anxious attachment and highly sociable dogs that are not prone to chase. Knowing that this attachment represents a tight, even clingy relationship between dog and owner, we may speculate that these dogs are used to closeness and proximity, resulting in also being more comfortable in the vicinity and company of other people.

By providing evidence of the associations between owner’s attachment style to pets and dog aggression, this study can serve as a foundation for future research on psychosocial factors affecting dog aggression. We believe that owners’ aggressive tendencies, dog training and socialization history and more in-depth exploration of the owner–dog bond are important psychosocial measures that can be further explored in the context of dog aggression. In this particular study, we used the personality taxonomy developed by Svartberg and Forkman [20] to investigate dog personality. However, there are other potentially useful measures (for review on dog personality assessment see Fratkin et al., [15]), yielding alternative dog personality traits that may potentially play a role in dog aggression.

## 5. Conclusions

Our results imply that both dogs’ and owners’ personality profiles predict dogs’ aggressive behavior. Similar to previous studies, neuroticism as the personality trait of an owner and sociability as the personality trait of a dog were closely associated with dogs exhibiting human-directed and animal-directed aggressive behavior. We first provided evidence suggesting that owners’ insecure attachment styles to pets, anxious and avoidant attachment, are linked to owner- and stranger-directed aggression in dogs, making owner–dog attachment style a potential predictor of undesired dog behavior. These results may contribute to the early detection of potentially dangerous traits, leading to better management and prevention of dog aggression towards humans, other dogs and other animals.

## Figures and Tables

**Table 1:** Differences between groups (Group 1 = non-aggressive dogs; Group 2 = dogs, aggressive towards humans; Group 3 = dogs, aggressive towards animals) in dogs’ personality traits. Bold—statistically significant result. Means with different superscript letters differ significantly.

Personality Trait	Group	Mean	SD	F-Value	*p*-Value
Playfulness	1	3.64	1.55	1.96	0.16
	2	2.15	1.68		
	3	3.59	1.83		
Curiosity	1	3.76	0.89	0.14	0.87
	2	3.58	0.78		
	3	3.56	0.74		
Chase-proneness	1	3.75	1.49	1.48	0.24
	2	2.42	1.57		
	3	3.58	1.69		
Sociability	1	3.79 ^a^	0.93	4.5	**0.02**
	2	2.58 ^b^	0.94		
	3	3.19 ^ab^	0.81		
Aggressiveness	1	2.11	0.68	0.73	0.49
	2	2.12	0.85		
	3	2.5	1.29		
Shyness/boldness	1	3.74	1.01	2.13	0.13
	2	3.69	1		
	3	3.48	1.09		

**Table 2:** Differences between groups (Group 1 = non-aggressive dogs; Group 2 = dogs, aggressive towards humans; Group 3 = dogs, aggressive towards animals) in owners’ personality traits. Bold—statistically significant result. Means with different superscript letters differ significantly.

Personality Trait	Group	Mean	SD	F-Value	*p*-Value
Extraversion	1	3.71	0.67	1.14	0.33
	2	3.23	1.01		
	3	3.69	1.07		
Agreeableness	1	3.68	0.64	0.82	0.45
	2	3.38	0.92		
	3	3.38	0.92		
Conscientiousness	1	3.79	0.82	0.02	0.98
	2	3.77	0.67		
	3	3.73	0.75		
Neuroticism	1	2.5 ^a^	0.89	3.85	0.03
	2	3.27 ^b^	0.88		
	3	2.5 ^a^	0.82		
Openness	1	3.46	0.79	0.31	0.74
	2	3.69	1.11		
	3	3.69	0.97		

**Table 3:** Significant correlations between attachment style, dog and owner personality traits and dog aggressive owner-reported behavior.

Attachment Style	Variable	r	*p*-Value
Anxious	Stranger-directed aggression	−0.4	0.01
	Chasing	−0.37	0.03
	Sociability	0.33	0.05
Avoidant	Owner-directed aggression	0.38	0.02
	Conscientiousness	−0.42	0.01

## Supplementary Materials

The following are available online at https://www.mdpi.com/2076-2615/10/2/315/s1, Table S1: Score sheet for DMA.
