# Supplementary material for: Dogs’ Sociability, Owners’ Neuroticism and Attachment Style to Pets as Predictors of Dog Aggression
Source: Animals (Basel). 2020 Feb 18;10(2):315. doi: 10.3390/ani10020315 (PMC7070865; doi:10.3390/ani10020315)
Supplement: Supplementary file 1 [file animals-10-00315-s001.zip › Table S1.docx]

**Supplementary Table**

**Table S1.** Score sheet for DMA.

| **Subtest** | **Behavioural variables** | **Score** | **Description of behaviour** |
| --- | --- | --- | --- |
| Social contact | Greeting reaction | 1 | Rejection of greeting |
|  |  | 3 | Interest in greeting |
|  |  | 5 | Intense greeting with jumping and whining |
|  | Cooperation | 1 | Refusal to walk with stranger |
|  |  | 3 | Willingness to walk with stranger, uncertain walk on a relaxed leash |
|  |  | 5 | High willingness to walk with stranger |
|  | Handling | 1 | Rejection of physical contact |
|  |  | 3 | Physical contact is not rejected, no social behaviour toward the stranger |
|  |  | 5 | Intense social behaviour towards stranger |
| Play 1 | Interest in play | 1 | No interest in the tossing of the rag |
|  |  | 3 | Interest in the tossing rag, but no following |
|  |  | 5 | Active play and following of the thrown rag |
|  | Grabbing | 1 | No grabbing |
|  |  | 3 | Interest in the rag, following, but no grabbing |
|  |  | 5 | Immediate and intense grabbing |
|  | Tug-of-war | 1 | No biting at all |
|  |  | 3 | Grabbing, but quick release of the rag |
|  |  | 5 | Immediate grabbing the rag with twitches and fighting until the TL releases the rag |
| Chase | Following 1 | 1 | No attempts to run after the fleeing object |
|  |  | 3 | Notice of the fleeing object and not immediate run after |
|  |  | 5 | Immediate reaction when seeing the object and running towards it with high speed |
|  | Grabbing 1 | 1 | No attempts to grab the object |
|  |  | 3 | Grabbing of the object after more than 3s |
|  |  | 5 | Immediate and intense grabbing combined with holding of the object in at least 3s |
|  | Following 2 | 1 | No attempts to run after the fleeing object |
|  |  | 3 | Notice of the fleeing object and not immediate run after |
|  |  | 5 | Immediate reaction when seeing the object and running towards it with high speed |
|  | Grabbing 2 | 1 | No attempts to grab the object |
|  |  | 3 | Grabbing of the object after more than 3s |
|  |  | 5 | Immediate and intense grabbing combined with holding of the object in at least 3s |
| Passive situation | Activity | 1 | Non-active |
|  |  | 3 | Active behaviour at the beginning of the subtest and non-active after, or vice versa |
|  |  | 5 | Active behaviour with switches between different modes of activity |
| Distance play | Aggression | 1 | No signs of aggression or threat display |
|  |  | 3 | Mild aggression display (quiet growling, low posture) |
|  |  | 5 | Threat display (growling, snarling, raised hackles, raised tail, etc.) directed against the assistant during both phase of threat and invitation |
|  | Exploration | 1 | No approach attempts towards the assistant, even when he is actively calling the dog |
|  |  | 3 | Approach after active calling |
|  |  | 5 | Immediate approach, even to the passive  assistant |
|  | Tug-of-war | 1 | No attempts to play tug-of-war |
|  |  | 3 | Play after invitation, passive grabbing of the rag |
|  |  | 5 | Immediate attempts to play with active pulling even when assistant is passive |
|  | Play invitation | 1 | No interest in the assistant |
|  |  | 3 | Interest in the assistant when he is active |
|  |  | 5 | Urgent play invitations from the dog to the assistant, even when he is passive |
| Sudden appearance | Startle reaction | 1 | A flight of > 5m |
|  |  | 3 | A flight of < 5m |
|  |  | 5 | Short hesitation |
|  | Aggression | 1 | No signs of aggression, or threat display |
|  |  | 3 | First reaction is attack against the dummy, no signs of aggression after |
|  |  | 5 | Threat display and attacks against dummy |
|  | Exploration | 1 | Great need of support (no approach of dummy until handler lowers it and sits close to it), or no approach |
|  |  | 3 | Need of support |
|  |  | 5 | Immediate approach to the dummy without need of support |
| Metallic noise | Startle reaction | 1 | A flight of > 5m |
|  |  | 3 | A flight of < 5m |
|  |  | 5 | Short hesitation |
|  | Exploration | 1 | No approach of the sheet of metal, even if handler sits close to it |
|  |  | 3 | Approach with handlers’ support |
|  |  | 5 | Immediate approach without need of support |
| Ghosts | Aggression | 1 | No signs of aggression or threat display |
|  |  | 3 | Threat display during the approach but not during the appearance |
|  |  | 5 | Threat displays and several attacks against the ghosts |
|  | Attention | 1 | Occasional glances towards the ghosts |
|  |  | 3 | Frequent staring towards ghosts |
|  |  | 5 | Constant staring and activity towards ghosts during the whole period of approaching |
|  | Exploration | 1 | No approach, at least not before step 4 |
|  |  | 3 | Approach during step 2 or 3 |
|  |  | 5 | Immediate approach after the dog is unleashed |
| Play 2 | Interest in play | 1 | No interest in the tossing of the rag |
|  |  | 3 | Interest in the tossing of the rag, but no following or play behaviour |
|  |  | 5 | Active play and following of the thrown rag |
|  | Grabbing | 1 | No grabbing |
|  |  | 3 | Staring towards the rag, occasional following |
|  |  | 5 | Immediate and intense grabbing |
